# The challenges of using new technologies (online education) for drowning prevention education in Iran

**Published:** 2022-12

**Authors:** Afzal Shamsi, Razia Shahabinejad, Milad Ahmadi Marzaleh, Saeed Nazari, Mansoureh Jafarkhani

**Affiliations:** ^*a*^ Department of Anesthesia and Emergency Medicine, School of Paramedical Sciences, Tehran University of Medical Sciences, Tehran, Iran.; ^*b*^ Arash Hospital, Tehran University of Medical Sciences, Tehran, Iran.; ^*c*^ Faculty of Medical Information and Management, Shiraz University of Medical Sciences, Shiraz, Iran.; ^*d*^ Department of Anesthesiology and Emergency Medicine, Tehran University of Medical Sciences, Tehran, Iran.; ^*e*^ Shafa Clinic, Education and Research Management, Nezaja Health, Relief and Treatment Department, Tehran, Iran.

## Abstract

**Background:**

Education is one of the most important components of the advanced world and human life. Virtual education can help increase learning motivation. Since the number of drownings is significant in Iran, this study was conducted to determine the challenges of using new technologies (online education) in the education of drowning prevention in the country.

**Methods:**

In this research, library resources were reviewed and Iranian databases related to the purpose of the study were searched.

**Results:**

The most important challenges related to the topic of the present study include internet restrictions in different parts of the country, broadband disruption, the newness of online education, weak management, poor teamwork, poor infrastructure in online education, insufficient number of experienced and interested teachers, etc.

**Conclusions:**

Using new technologies, especially online education to teach drowning prevention in Iran has faced many challenges which can be resolved. Therefore, it is necessary to plan on eliminating these problems and strengthening drowning education.

**Keywords:**

Education, Online Education, Immersion

